# Discrepancy between the Radiographic Screening by Magnetic Resonance Imaging and Histopathologic Diagnosis of Vascular Malformations

**DOI:** 10.3400/avd.oa.26-00079

**Published:** 2026-08-20

**Authors:** Makoto Shiraishi, Kou Fujisawa, Yoichi Yasunaga, Yuta Moriwaki, Yukako Shibasaki, Haesu Lee, Tetsuo Ushiku, Osamu Abe, Mutsumi Okazaki

**Affiliations:** 1 Department of Plastic and Reconstructive Surgery, Graduate School of Medicine, The University of Tokyo, Tokyo, Japan; 2 Department of Pathology, Graduate School of Medicine, The University of Tokyo, Tokyo, Japan; 3 Department of Radiology, Graduate School of Medicine, The University of Tokyo, Tokyo, Japan

**Keywords:** vascular malformations, magnetic resonance imaging, arteriovenous malformation, venous malformation, lymphatic malformation

## Abstract

**Objectives:**

Although magnetic resonance imaging (MRI) is widely used to evaluate extracranial vascular anomalies (VAs), its diagnostic accuracy remains uncertain.

**Methods:**

We retrospectively assessed MRI accuracy for VAs and identified nonvascular lesions mimicking them. Patients who underwent surgical resection for suspected VAs at a tertiary referral center in Japan between April 2019 and June 2025 were analyzed.

**Results:**

A total of 172 patients met the inclusion criteria (mean age 34.9 ± 18.8 years; 62.8% female). Lesions were most commonly located in the extremities (54.1%), followed by the head and neck (32.6%) and the trunk (13.4%). Histopathology identified venous malformations (VMs) as the most frequent subtype (73.8%), followed by arteriovenous malformations (AVMs; 11.0%) and lymphatic malformations (LMs; 6.4%). MRI showed moderate sensitivity and high specificity: AVM sensitivity 0.68 and specificity 0.99; VM sensitivity 0.70 and specificity 0.98; LM sensitivity 0.80 and specificity 0.98. Seven cases (4.1%) were ultimately diagnosed as non-VA lesions despite MRI findings suggestive of VAs. Common mimickers included angioleiomyoma and myopericytoma.

**Conclusions:**

MRI demonstrates moderate sensitivity and strong specificity for VAs, although false-positive interpretations remain an important consideration.

## Abbreviations

ISSVA
: International Society for the Study of Vascular Anomalies
VAs
: vascular anomalies
MRI
: magnetic resonance imaging
AVM
: arteriovenous malformation
VM
: venous malformation
LM
: lymphatic malformation
PPV
: positive predictive value
NPV
: negative predictive value

## Introduction

Extracranial vascular anomalies (VAs), including hemangiomas and vascular malformations, require specialized care at centers with multidisciplinary collaboration.^1,2)^ Accurate diagnosis, classified by the International Society for the Study of Vascular Anomalies (ISSVA), is critical for determining optimal therapeutic strategies.^3)^ Along with ultrasound, magnetic resonance imaging (MRI) plays a pivotal role as the primary screening tool for characterizing vascular lesions prior to surgical intervention. Most patients with suspected VAs are referred to tertiary medical centers based on MRI findings. Nevertheless, the diagnostic precision of MRI for VAs remains less explored than that for intracranial or cerebral vascular pathologies.^4,5)^

Although numerous reports have addressed the imaging characteristics of MRI for intracranial malformations and hepatic hemangiomas, there is limited literature on lesions.^6–12)^ The anatomical variability and tissue composition of cutaneous and soft tissue regions often complicate radiological interpretation. Furthermore, certain benign soft tissue tumors, reactive lesions, inflammatory processes, and fibrotic conditions may closely mimic VAs, substantially increasing the risk of misdiagnosis.^13,14)^ As a result, the MRI criteria developed for intracranial lesions may not be directly applicable to cutaneous and soft tissue cases. Therefore, a systematic investigation of the diagnostic performance of MRI for VAs is warranted to guide clinicians in refining diagnostic approaches and optimizing surgical planning.

Complete surgical resection remains the definitive treatment for many VAs, including arteriovenous malformations (AVMs), venous malformations (VMs), and lymphatic malformations (LMs).^15–20)^ However, histopathologic examination occasionally reveals non-VA lesions, such as angioleiomyoma or glomus tumor, that were radiologically misinterpreted as VAs preoperatively.^4)^ These findings underscore a clinically significant diagnostic “gap” between preoperative radiologic assessment and definitive histopathologic confirmation. Few studies have systematically analyzed the MRI characteristics of non-VA lesions that mimic VAs.

To address these knowledge gaps, we conducted a retrospective cohort study to evaluate (1) the diagnostic accuracy of MRI for VAs, including the sensitivity, specificity, and predictive values for different VA subtypes, and (2) the demographic, anatomical, and pathological characteristics of non-VA lesions that radiologically mimic VAs.

## Materials and Methods

### Study design and setting

This single-center retrospective cohort study was conducted at the University of Tokyo Hospital in Japan, an academic tertiary referral center, from April 1, 2019, to June 30, 2025. The study protocol was approved by the Institutional Review Board of the University of Tokyo Hospital (approval number: 2020315). All procedures were performed in accordance with the ethical standards of the Institutional Research Committee and the 1964 Declaration of Helsinki and its later amendments. Instead of obtaining informed consent from patients, this study applied an opt-out system on the official website of the University of Tokyo Hospital. The present study adhered to the STROBE guidelines.

### Study population

Patients were considered eligible for inclusion if they had undergone surgical resection for suspected VAs, including hemangiomas and VMs, during the study period. All patients underwent preoperative MRI at our institution or at the referring hospitals. Patients who underwent biopsy before definitive surgical resection were excluded from this study. Data were collected from medical records at the time of surgical treatment and included patient age at treatment, sex, anatomical location of the lesion, preoperative radiological diagnosis based on MRI findings, and the final histopathological diagnosis.

### MRI protocol

MRI was performed using various scanners depending on the referring institution or imaging facility. Standard imaging protocols for suspected low-flow vascular malformations included T1- and T2-weighted spin-echo sequencing. For suspected high-flow VMs, additional magnetic resonance angiography and magnetic resonance venography were obtained to assess vascular architecture and evaluate the feasibility of preoperative embolization. Non-dynamic (non-contrast-enhanced) MRI sequences were used for the initial diagnostic assessment in all cases. The preoperative diagnosis was defined as the radiological impression documented in the official clinical MRI reports by board-certified radiologists. This approach was adopted deliberately to assess the diagnostic accuracy of MRI reports as they are actually issued and used to guide referral and treatment in routine clinical practice, rather than under idealized re-reading conditions.

### Diagnostic criteria and classification

**Fig. 1:**
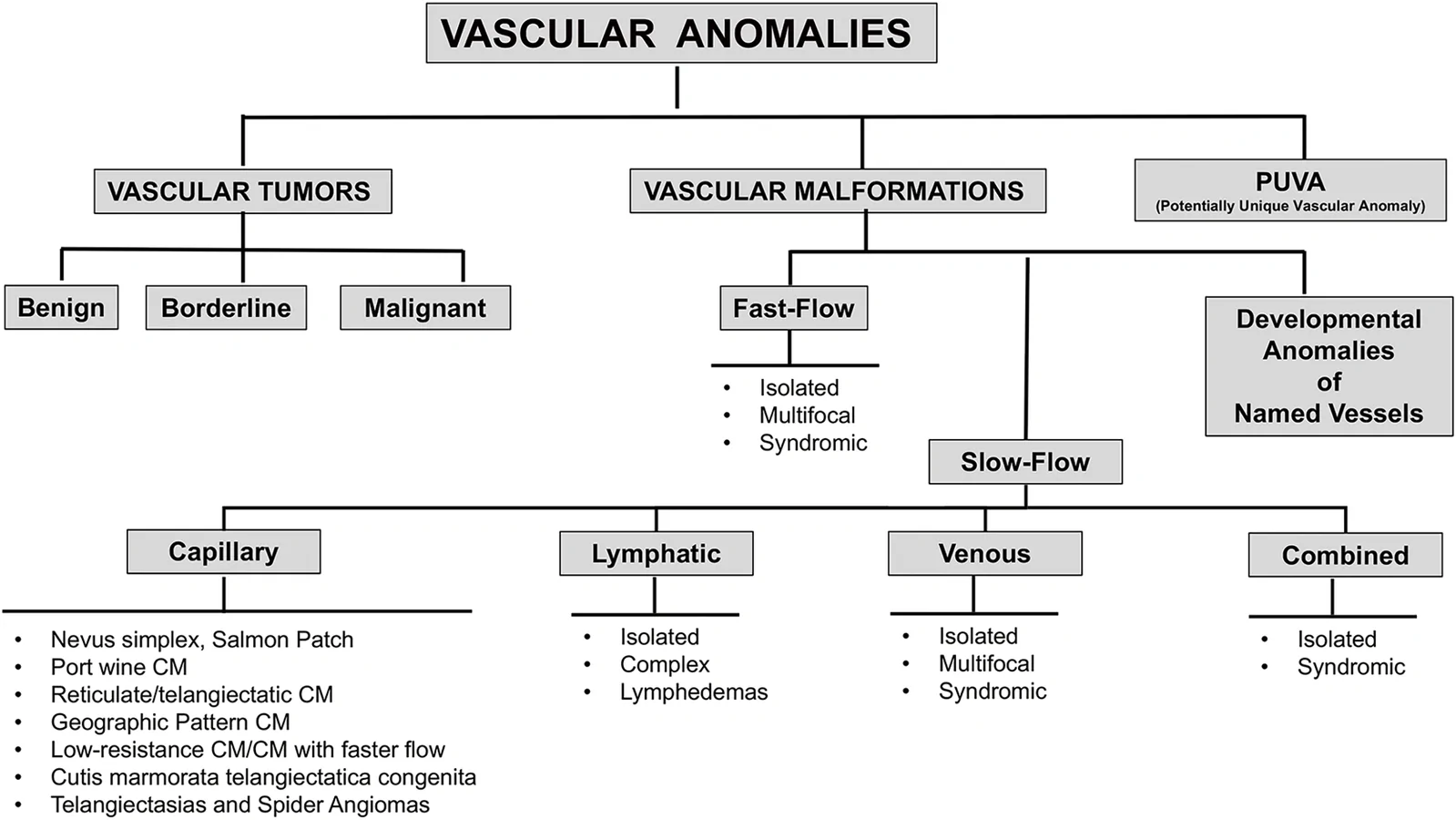
Updated ISSVA classification in 2025. ISSVA classification of vascular anomalies. Reproduced from Ref. 3 with permission. ©2025 International Society for the Study of Vascular Anomalies. CM, capillary malformation

VAs were classified according to the ISSVA classification system (**Fig. 1**).^3)^ Preoperative MRI diagnoses were compared with the final histopathological diagnoses obtained from the surgical specimens. Histopathological examinations were performed by experienced pathologists specializing in vascular lesions. Cases were categorized as true VAs or nonvascular anomaly (non-VA) lesions based on the final pathological diagnosis.

### Statistical analysis

All statistical analyses were performed using SPSS version 29.0 (IBM, Armonk, NY, USA). The distribution of continuous variables was assessed for normality, and these variables are reported as mean ± standard deviation. Categorical data are expressed as counts and percentages.

The diagnostic accuracy of MRI was examined by calculating sensitivity, specificity, positive predictive value (PPV), and negative predictive value (NPV) for each subtype of vascular anomaly (arteriovenous, venous, and LMs) in a univariate manner. These indices were derived from the numbers of true positives (TP), true negatives (TN), false positives (FP), and false negatives (FN). Sensitivity was defined as TP/(TP+FN), and specificity as TN/(TN+FP). PPV and NPV were calculated as TP/(TP+FP) and TN/(TN+FN), respectively, with PPV being influenced by disease prevalence. Exact binomial 95% confidence intervals (95% CI) were calculated for sensitivity, specificity, PPV, and NPV.

The chi-square test was used to compare categorical variables, including diagnostic accuracy, between different subgroups. Adjusted standardized residuals were computed to identify cells in the cross-tabulations where the observed frequencies were significantly different from the expected frequencies. Positive adjusted standardized residuals greater than 1.96 indicated significantly more participants than expected, whereas negative adjusted standardized residuals less than –1.96 indicated significantly fewer participants than expected. A 2-tailed P-value <0.05 was considered statistically significant for all analyses.

## Results

In total, 172 patients with suspected VAs who underwent surgical resection were included in this study (**Table 1**). The mean age at treatment was 34.9 ± 18.8 years. The cohort comprised 108 females (62.8%) and 64 males (37.2%), with a female-to-male ratio of 1.7:1. A total of 167 participants were Japanese (97.0%).

**Table 1:** Demographics of the subjects (N = 172)

Item	N	Percent
Number of patients	172	100%
Age, mean (SD)	34.9 (18.8)	
Sex		
Male	64	37.2%
Female	108	62.8%
Ethnicity		
Japanese	167	97.0%
Location of VAs		
Head and neck	56	32.6%
Extremities	93	54.1%
Trunk	23	13.4%
Pathologic diagnosis		
VM	127	73.8%
AVM	19	11.0%
LM	9	6.4%
LVM	3	1.2%
Other types of VAs	10	5.8%
Non-VAs	7	4.1%

SD, standard deviation; VAs, vascular anomalies (including hemangioma and vascular malformations according to ISSVA Classification); AVM, arteriovenous malformation; VM, venous malformation; LM, lymphatic malformation; LVM, lymphatic venous malformation; ISSVA, International Society for the Study of Vascular Anomalies

Regarding anatomical distribution, the majority of lesions were located in the extremities (n = 93, 54.1%), followed by the head and neck region (n = 56, 32.6%) and the trunk (n = 23, 13.4%). Among the final histopathological diagnoses, VMs were the most common, accounting for 127 cases (73.8%), followed by AVMs in 19 cases (11.0%) and LMs in 9 cases (6.4%).

The diagnostic performance of MRI varied across different subtypes of VAs (**Table 2**). For AVMs, MRI demonstrated a sensitivity of 0.68 (95% CI: 0.44–0.87), specificity of 0.99 (95% CI: 0.96–0.99), PPV of 0.93 (95% CI: 0.64–0.99), and NPV of 0.96 (95% CI: 0.93–0.98). For VMs, the sensitivity of MRI was 0.70 (95% CI: 0.61–0.78), with a specificity of 0.98 (95% CI: 0.87–0.99), PPV of 0.99 (95% CI: 0.93–0.99), and NPV of 0.51 (95% CI: 0.45–0.58). Regarding LMs, MRI showed a sensitivity of 0.80 (95% CI: 0.44–0.97), specificity of 0.98 (95% CI: 0.95–0.99), PPV of 0.73 (95% CI: 0.45–0.90), and NPV of 0.99 (95% CI: 0.96–0.99).

**Table 2:** Diagnostic accuracy of MR imaging for soft tissue VAs

	Prevalence (95% CI)	Sensitivity (95% CI)	Specificity (95% CI)	Positive predictive value (95% CI)	Negative predictive value (95% CI)
AVM	0.11 (0.07–0.17)	0.68 (0.44–0.87)	0.99 (0.96–0.99)	0.93 (0.64–0.99)	0.96 (0.93–0.98)
VM	0.75 (0.68–0.82)	0.70 (0.61–0.78)	0.98 (0.87–0.99)	0.99 (0.93–0.99)	0.51 (0.45–0.58)
LM	0.06 (0.03–0.10)	0.80 (0.44–0.97)	0.98 (0.95–0.99)	0.73 (0.45–0.90)	0.99 (0.96–0.99)

^*^P <0.05, ^**^P <0.005, ^***^P <0.001.

VAs, vascular anomalies (including hemangioma and vascular malformations according to ISSVA Classification); AVM, arteriovenous malformation; VM, venous Malformation; LM, lymphatic malformation

A total of 7 of the 172 cases (4.07%) were pathologically confirmed as nonvascular anomaly lesions despite preoperative MRI interpretation suggesting VA (**Table 1**). The distribution of these non-VA lesions was as follows: angioleiomyoma was the most common mimicker, accounting for 3 cases (1.74% of the total cohort; **Supplementary Fig. S1**), followed by myopericytoma (n = 1, 0.58%; **Supplementary Fig. S2**), glomus tumor (n = 1, 0.58%), neurofibroma (n = 1, 0.58%), and pleomorphic adenoma (n = 1, 0.58%) (**Table 3**). The detailed clinical and imaging characteristics of each misdiagnosed case are presented in **Supplementary Table S1**.

**Table 3:** Summary of misdiagnosed cases as VAs, N = 172

Pathological diagnosis	N	%
Angioleiomyoma	3	1.74
Myopericytoma	1	0.58
Glomus tumor	1	0.58
Neurofibroma	1	0.58
Pleomorphic adenoma	1	0.58

VAs, vascular anomalies (including hemangioma and vascular malformations)

## Discussion

In this study, we analyzed 172 patients who underwent surgical resection for VAs to assess the diagnostic accuracy of MRI. Most lesions were VMs, followed by arteriovenous and LMs. MRI demonstrated moderate sensitivity (0.68–0.80) and high specificity (0.98–0.99) across all subtypes of VAs. Pathological examination revealed that 4.1% of lesions were ultimately diagnosed as non-VAs, with angioleiomyoma and myopericytoma being the most frequent mimickers. These findings indicate that several benign cutaneous and soft tissue tumors can mimic VMs on MRI, contributing to diagnostic challenges in clinical practice. Collectively, these results highlight both the strengths and limitations of MRI in differentiating VAs from their mimickers. To the best of our knowledge, this is one of the first comprehensive studies to systematically evaluate the diagnostic accuracy of MRI for VAs.

Numerous studies have evaluated the diagnostic performance of MRI for intracranial AVMs and hepatic hemangiomas, and few reports have specifically addressed VAs. For brain AVMs, the reported sensitivity ranges from 80% to 95%, with a specificity of nearly 100%, whereas for hepatic hemangiomas, the reported sensitivity ranges from 92% to 100%, with specificity between 86.0% and 93.0%.^6–11)^ In contrast, we observed moderate sensitivity (0.68–0.80) and high specificity (0.98–0.99) for VAs. This diagnostic pattern observed in our study may be explained by the clinical context in which MRI is used. MRI is often performed for initial screening purposes, and radiologists tend to report all possible diagnoses to avoid missing significant vascular lesions, which subsequently leads to referrals to tertiary care centers. A similar trend has been observed in breast cancer screening programs. Hayward et al.^12)^ reported that the specificity of MRI for initial screening was 55%, which improved to 83% in subsequent follow-up screening, suggesting that conservative interpretation strategies during initial evaluation contribute to higher false-positive rates, but ensure that fewer true cases are missed. In addition, a relatively low PPV (0.51) was observed compared to AVM (0.96) and LM (0.99), but this trend might be due to the relatively higher prevalence of VM (75%) than that of AVM (11%) and LM (6%).

Because MRI alone demonstrated only moderate sensitivity, combining it with complementary imaging is likely to improve lesion detection. Doppler ultrasonography, in particular, provides real-time hemodynamic information that reliably distinguishes high-flow from low-flow lesions and can characterize small or superficial lesions that are difficult to resolve on MRI. The current Japanese clinical practice guidelines accordingly recommend ultrasonography in conjunction with MRI as first-line imaging for VAs.^20)^ Integrating Doppler ultrasonography with MRI may therefore compensate for the moderate sensitivity observed in the present study and reduce the risk of missed or misclassified lesions.

To date, few studies have systematically addressed the discrepancies between MRI findings and final pathological diagnoses of VAs. Several case reports have identified tumors that mimic VAs on imaging. Lo et al.^21)^ reported a case of alveolar soft part sarcoma of the thigh that was initially misdiagnosed as an AVM. Hayek et al.^13)^ described 2 cases of infantile fibrosarcoma in the upper extremities that were misdiagnosed as AVMs. Additionally, Armstrong et al.^14)^ presented a case of a foreign body that mimicked a low-flow VM on MRI. In cases misdiagnosed as AVMs, as reported by Lo et al. and Hayek et al., malignant pathology was ultimately identified because blood flow-rich lesions can demonstrate vascular flow patterns similar to those of true VAs.^13,21)^ In contrast, in our cohort, all pathologically confirmed non-VA lesions exhibited rich blood flow on imaging, but none proved to be malignant on final pathological examination.

The existence of these diagnostic mimickers underscores the importance of maintaining clinical vigilance, particularly when imaging features are atypical or when lesions fail to respond to treatment as expected. Horbach et al.^4)^ conducted a retrospective study of 142 cases to evaluate the concordance between the clinical and histological diagnoses of VAs. In their cohort, at least 1 imaging modality was performed in 83.9% of patients, and MRI was obtained in 63.6%. Preoperative imaging was negatively associated with a complete discrepancy between the clinical and final histological diagnoses (P = 0.04), suggesting that imaging improves diagnostic accuracy. Conversely, diagnostic uncertainty among clinicians was identified as a significant risk factor for diagnostic discrepancies (P = 0.003). These findings highlight the importance of a comprehensive preoperative evaluation. When there is any suspicion of malignancy based on the clinical presentation or imaging features, multiple imaging modalities should be employed, and immediate consultation with oncology specialists is warranted, as malignancy must always be considered in the differential diagnosis of suspected vascular lesions.

From a management perspective, the clinical relevance of an accurate preoperative diagnosis extends beyond the distinction between benign and malignant disease. Although most lesions in our cohort were benign, an incorrect assignment of the vascular anomaly subtype can still result in inappropriate treatment selection. For example, a mimicker such as a retained foreign body that is misdiagnosed as a VMs may be treated with sclerotherapy, which is ineffective for such lesions and exposes the patient to unnecessary risk and delay; conversely, correctly recognizing a lesion as a nonvascular tumor prompts complete surgical excision with appropriate histopathological evaluation.^14)^ In our institution, surgical decisions were not based on MRI findings alone but on the integration of clinical presentation, imaging, and, when indicated, additional modalities; nonetheless, MRI remained the principal modality directing referral and the initial treatment strategy. These observations indicate that the diagnostic accuracy of MRI can directly affect the appropriateness of the management plan, underscoring the clinical value of recognizing vascular anomaly mimickers preoperatively.

This study has several strengths. First, this is one of the few long-term studies that specifically focused on the diagnostic performance of MRI for VAs. Second, the study included a relatively large cohort of 172 patients, all of whom were surgically treated at a single tertiary referral center, which allowed for the standardization of surgical techniques and pathological assessment protocols. However, we also acknowledge some important limitations. First, due to the retrospective nature of the study, potential recall and selection biases may have been present. In particular, because only patients who underwent surgical resection were included, lesions managed conservatively or treated solely with sclerotherapy or embolization were not captured, which may have shifted the lesion spectrum toward those considered surgical candidates. We nevertheless regarded surgical resection as an indispensable inclusion criterion, because a definitive histopathological reference standard—and therefore a valid evaluation of the diagnostic accuracy of MRI—cannot be obtained without a resected specimen. Restricting the cohort to surgically resected cases was thus a deliberate trade-off that prioritized diagnostic certainty over a fully representative lesion spectrum. Second, the MRI protocols varied among referral hospitals, which may have affected the consistency and comparability of imaging interpretations and could have influenced the overall diagnostic accuracy. Third, the study was conducted at a single academic center with a specific referral population, and both preoperative and postoperative management followed institution-specific protocols. Therefore, our findings may not be directly generalizable to other clinical settings or healthcare systems. Nevertheless, the single-center approach also conferred the advantage of standardizing treatment regimens and minimizing variability in surgical and pathological evaluations, thereby enhancing the internal validity of our findings. Future multicenter studies with standardized imaging protocols would be valuable to confirm and extend these findings to broader clinical contexts. Fourth, 97% of participants were Japanese, limiting applicability to other ethnicities. Finally, genetic testing was not conducted, as these tests are not well established or covered by the national health insurance in Japan.

## Conclusion

MRI has moderate sensitivity (0.68–0.80) for detecting VAs but has high specificity (0.98–0.99), however, suspected VAs by MRI may represent alternative diagnoses like angioleiomyoma and myopericytoma. Given the moderate sensitivity and the diagnostic challenges posed by VA mimickers, combining MRI with Doppler ultrasonography, together with multidisciplinary collaboration, is warranted, particularly in ambiguous cases. Future studies should identify specific MRI features to improve differentiation and optimize diagnostic algorithms.

## Supplementary Materials

Supplementary Fig. S1 A case of angioleiomyoma of the lower lip misdiagnosed as VM (Case #2 of Supplementary Table S1). (**A**) A 62-year-old male presented with a subcutaneous mass on the right plantar region referred from a community hospital with MR images. (**B**) T2-weighted MR image. (**C**) STIR MR image. (**D**) Histopathologically (hematoxylin and eosin staining), smooth muscle cells with small, uniform nuclei and eosinophilic fibrillar cytoplasm proliferate surrounding muscular vessels of various sizes or forming the vessel walls, creating a well-circumscribed lobulated mass, which consequently represents an angioleiomyoma.

Supplementary Fig. S2 A case of myopericytoma of the right plantar region misdiagnosed as VM (Case #4 of Supplementary Table S1). (**A**) A 62-year-old female presented with a subcutaneous mass on the right plantar region referred from a community hospital with MR images. (**B**) Fat-suppressed T2-weighted MR image. (**C**) Fat-suppressed T1-weighted MR image. (**D**) Histopathologic examination (hematoxylin and eosin staining) revealed a dilated vein measuring approximately 5 mm in the subcutaneous tissue, with spindle-shaped tumor cells exhibiting ovoid nuclei without significant atypia proliferating concentrically around blood vessels, consistent with myopericytoma.

Supplementary Table S1 Descriptions of participants pathologically diagnosed with non-soft tissue vascular malformations.

## Declarations

### Data availability statement

The data that support the findings of this study are available from the corresponding author upon reasonable request.
