# Supplementary material for: Discrepancy between the Radiographic Screening by Magnetic Resonance Imaging and Histopathologic Diagnosis of Vascular Malformations
Source: Ann Vasc Dis. 2026 Aug 20;19(1):26-00079. doi: 10.3400/avd.oa.26-00079 (PMC13494577; doi:10.3400/avd.oa.26-00079)

Supplementary Fig. S1

A B C D

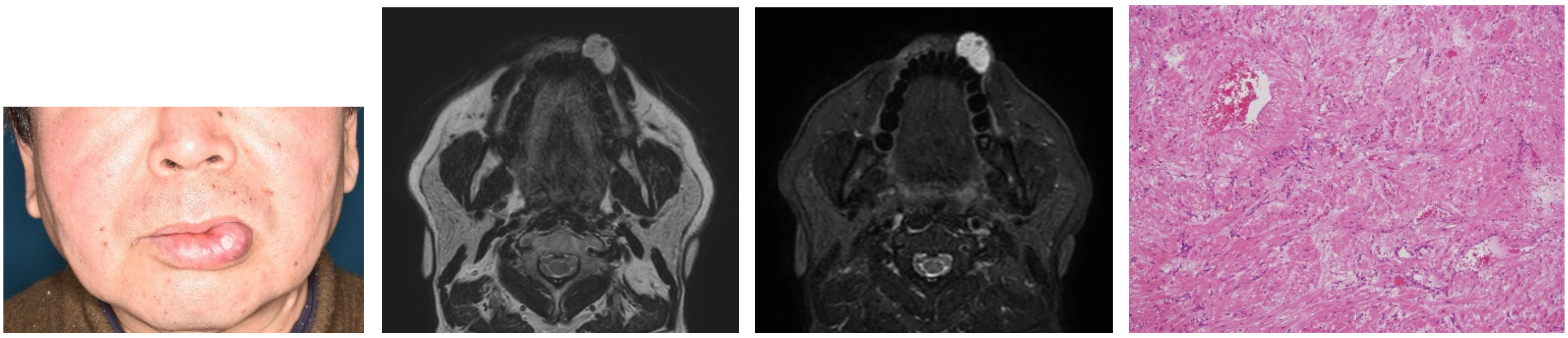

Supplementary Fig. S2

A B C D

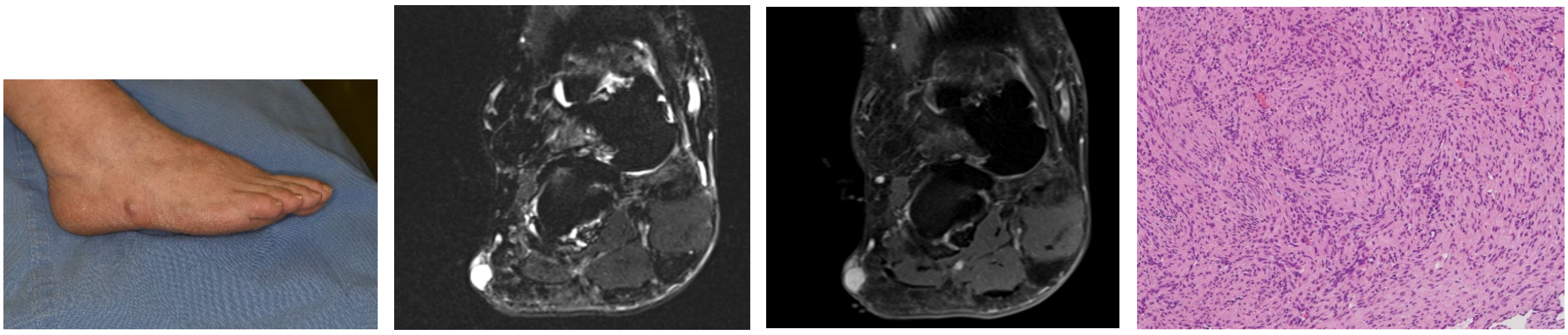

Supplement: Supplementary file 1 [file avd-19-1-26-00079-s001.pdf]
