# Supplementary material for: Discrepancy between the Radiographic Screening by Magnetic Resonance Imaging and Histopathologic Diagnosis of Vascular Malformations
Source: Ann Vasc Dis. 2026 Aug 20;19(1):26-00079. doi: 10.3400/avd.oa.26-00079 (PMC13494577; doi:10.3400/avd.oa.26-00079)
Supplement: Supplementary file 2 [file avd-19-1-26-00079-s002.pdf]

**Supplementary Table S1** Descriptions of participants pathologically diagnosed with non-soft tissue vascular malformations, N = 172

| Case # | Sex | Age at initial treatment | Location | Preoperative Diagnosis | Pathological Diagnosis | Treatment |
|---|---|---|---|---|---|---|
| 1 | M | 56 | Lt. finger | AVM | Angioleiomyoma | Resection + Free Flap |
| 2 | M | 62 | Lower lip | VM | Angioleiomyoma | Resection |
| 3 | M | 54 | Lt. ear | VM | Angioleiomyoma | Resection |
| 4 | F | 62 | Lt. plantar | VM | Myopericytoma | Resection |
| 5 | M | 44 | Rt. upper limb | VM | Glomus Tumor | Resection |
| 6 | F | 38 | Lt. pelvis | VM | Neurofibroma | Resection |
| 7 | M | 53 | Lt. cheek | LM | Pleomorphic Adenoma | Resection |

M, male; F, female; Rt., right; Lt., left; AVM, arteriovenous malformation; VM, venous malformation; LM, lymphatic malformation; Vas, vascular anomalies (including hemangioma and vascular malformations)
